# Supplementary material for: Implementation of Pelvic Floor Rehabilitation after rectal cancer surgery: A qualitative study guided by the Consolidated Framework for Implementation Research (CFIR)
Source: PLoS One. 2024 Jun 20;19(6):e0301518. doi: 10.1371/journal.pone.0301518 (PMC11189208; doi:10.1371/journal.pone.0301518)
Supplement: S1 File — (DOCX) [file pone.0301518.s001.docx]

**Supplementary file 1.**
Patient information letter prior to referral to PFR, as provided by the FORCE trial.

Dear Mr./Mrs.,

Unfortunately, you have recently received a diagnosis of rectal cancer. As a result, you will need to undergo surgery in the near future. In this brochure, we will provide you with information about the potential consequences of rectal cancer surgery.

During the surgery, a part of the rectum will be removed, which will have implications for your defecation pattern. The rectum serves as a reservoir where bowel movements are collected, and when the rectum is smaller, the frequency of bowel movements will increase. After the surgery, many individuals experience changes in their defecation pattern, such as increased bowel movements, a heightened urge to defecate, diarrhea, fecal incontinence, or difficulty in emptying the bowel. While these changes can impact your daily life, most of these symptoms will improve in the following weeks. They can be managed with medications that alter stool composition, making it easier to control bowel movements, or with anti-diarrheal medicines. Adjusting your liquid and/or fiber intake may help alleviate the symptoms.

If you continue to experience fecal incontinence, an increased number of bowel movements, or urgency, pelvic floor physiotherapy can be beneficial in improving your symptoms. Pelvic floor physiotherapy involves training the pelvic floor muscles combined with biofeedback, electrostimulation, and rectal balloon training.

**Pelvic Floor Training with Biofeedback, Electrostimulation, and Rectal Balloon Training**A specialized physiotherapist will work with you to enhance the function of your pelvic floor muscles. These muscles consist of three layers and provide support to the internal organs. When you contract the pelvic floor muscles, they lift the internal organs in the pelvis and tighten the openings of the vagina, anus, and urethra. Relaxing the pelvic floor allows for the passage of urine and feces. Pelvic floor muscles can be either too tight or too weak, which can lead to problems like fecal incontinence or obstructed defecation. Since these muscles are not visible or touchable, as they are hidden within the pelvis, you can only feel them when you contract or relax them.

Biofeedback is used to visualize the activity of the pelvic floor muscles and provide direct feedback during exercises. A small electrode with sensors will be inserted into your anal canal to measure the activity of the pelvic floor layers. This information will help guide you in performing the appropriate exercises for your pelvic floor muscles. Training the pelvic floor muscles requires them to contract quickly and briefly while maintaining endurance. Relaxation and coordination of these muscles are also crucial. The training will be practiced in various positions, including lying on your side, sitting, and standing, to simulate daily life situations.

Electrostimulation is employed to strengthen and improve the effectiveness of pelvic floor muscle contractions. It is particularly useful when contractions of the pelvic floor muscles are not easily observable or palpable. Electrostimulation can be utilized during all pelvic floor physiotherapy sessions with biofeedback, using the same anal probe.

**Rectal Balloon Training**

Rectal balloon training is designed to simulate the sensation of needing to defecate. During this training, a rectal balloon will be inserted into the neo-rectum (the remaining part of the rectum). Using a syringe connected to the balloon, the balloon will be slowly inflated. You will be instructed to pay attention to your sensations of rectal filling, and when you feel a strong desire to defecate, you should produce an adequate contraction of the sphincter and puborectal muscle to contain the balloon. This training helps reduce the urge to defecate and increases confidence in maintaining fecal continence.
