# Supplementary material for: Implementation of Pelvic Floor Rehabilitation after rectal cancer surgery: A qualitative study guided by the Consolidated Framework for Implementation Research (CFIR)
Source: PLoS One. 2024 Jun 20;19(6):e0301518. doi: 10.1371/journal.pone.0301518 (PMC11189208; doi:10.1371/journal.pone.0301518)
Supplement: S2 File — (DOCX) [file pone.0301518.s002.docx]

**Supplementary file 2.**

A list of items suggested to be included in the referral letter to PFR.

1. Name:……………………………………………………….
2. Date of birth:………………………………………………..
3. Date of surgery:…………………………………………….

| Surgery | LAR TME TEM APR |
| --- | --- |
| Complications | Anastomose leakage: Yes / No  Other: ……………………………………………………………………………………… ……………………………………………………………………………………………… ……………………………………………………………………………………………… |
| Anastomosis height | …… cm above anal verge |
| Medical restriction anal probe/rectal balloon | Yes / No |
| Name and signature |  |
