# Supplementary material for: Implementation of Pelvic Floor Rehabilitation after rectal cancer surgery: A qualitative study guided by the Consolidated Framework for Implementation Research (CFIR)
Source: PLoS One. 2024 Jun 20;19(6):e0301518. doi: 10.1371/journal.pone.0301518 (PMC11189208; doi:10.1371/journal.pone.0301518)
Supplement: Attachment — Submitted filename: Response to Reviewers.docx [file pone.0301518.s003.docx]

**Response to Editorial Board**

**Question:** 1. Please ensure you have included the registration number for the clinical trial referenced in the manuscript.

**Answer:** This registration number is added in the manuscript.

**Question:** 2. We note that the grant information you provided in the ‘Funding Information’ and ‘Financial Disclosure’ sections do not match. When you resubmit, please ensure that you provide the correct grant numbers for the awards you received for your study in the ‘Funding Information’ section.
**Answer:** There was no funding for this study, although the original (FORCE) trial itself was funded by the Netherlands Organization for Health Research and Development (ZonMw, file number 80-84300-98-72021). The funder has had no role in the conceptualization, design, data collection, analysis, decision to publish, or preparation of the manuscript.

Somehow I cannot change this in the digital manuscript submission system, which is why I have changed this in the manuscript. Hope this is sufficient for you at this moment in time.

**Question** 3. Thank you for updating your data availability statement. You note that your data are available within the Supporting Information files, but no such files have been included with your submission. At this time we ask that you please upload your minimal data set as a Supporting Information file, or to a public repository such as Figshare or Dryad. Please also ensure that when you upload your file you include separate captions for your supplementary files at the end of your manuscript. As soon as you confirm the location of the data underlying your findings, we will be able to proceed with the review of your submission.
**Answer:** The data from is now fully available via an online repository. <https://doi.org/10.17026/dans-xmk-twgb>

**Question:** 4. Please include your tables as part of your main manuscript and remove the individual files. Please note that supplementary tables (should remain/ be uploaded) as separate ""Supporting Information"" files
**Answer:** We have adjusted this.

**Question:** 5. Please upload a copy of Figure 1 which you refer to in your text. Or if the figure is no longer to be included as part of the submission please remove all reference to it within the text.
**Answer:** We have adjusted this.

**Response to Reviewers**

**Question/request.
1. Please ensure that your manuscript meets PLOS ONE's style requirements, including those for file naming. The PLOS ONE style templates can be found at**

[**https://journals.plos.org/plosone/s/file?id=wjVg/PLOSOne_formatting_sample_main_body.pdf**](https://journals.plos.org/plosone/s/file?id=wjVg/PLOSOne_formatting_sample_main_body.pdf)

[**https://journals.plos.org/plosone/s/file?id=ba62/PLOSOne_formatting_sample_title_authors_affiliations.pdf**](https://journals.plos.org/plosone/s/file?id=ba62/PLOSOne_formatting_sample_title_authors_affiliations.pdf)

**Answer:** We have made several adjustments to adhere to the style requirements.

**Question/request.
2. Please ensure you have included the registration number for the clinical trial referenced in the manuscript.**

**Answer:** The registration number for the clinical trial is now referenced in the methods section.

**Question/request.
3. We note that the grant information you provided in the ‘Funding Information’ and ‘Financial Disclosure’ sections do not match. When you resubmit, please ensure that you provide the correct grant numbers for the awards you received for your study in the ‘Funding Information’ section.**

**Answer:** we have adjusted this.

**Question/request.
4. Your ethics statement should only appear in the Methods section of your manuscript. If your ethics statement is written in any section besides the Methods, please move it to the Methods section and delete it from any other section. Please ensure that your ethics statement is included in your manuscript, as the ethics statement entered into the online submission form will not be published alongside your manuscript.**

**Answer:** The ethics statement is removed from the ‘declarations’ section and now appears only in the methods section of our manuscript.

**Question/request.**

**5. Please review your reference list to ensure that it is complete and correct. If you have cited papers that have been retracted, please include the rationale for doing so in the manuscript text, or remove these references and replace them with relevant current references. Any changes to the reference list should be mentioned in the rebuttal letter that accompanies your revised manuscript. If you need to cite a retracted article, indicate the article’s retracted status in the References list and also include a citation and full reference for the retraction notice.**
**Answer:** We did not have to make changes.

Reviewers' comments:

**Comments to the Author**

Please use the space provided to explain your answers to the questions above. You may also include additional comments for the author, including concerns about dual publication, research ethics, or publication ethics. (Please upload your review as an attachment if it exceeds 20,000 characters)

**Qestion/request:
Reviewer #1:** Thank you for providing this opportunity for me to review this good study. This research work is very well executed. However this research was conducted between October 2017 and March 2020. **In 2022, an updated version of the CFIR has been released with relatively large changes based on feedback received from CFIR users. According to the publication of this article, it does not have the necessary aspect of novelty in this journal.**

Answer: Dear reviewer 1, you are correct. The research in which the participants were included was conducted between October 2017 and March 2020. However, the final version of this manuscript (which is a follow-up study of the original trial which ended inclusion in March 2020) already used the updated version of the CFIR as described in the article from Damschroder et al, 2022 (implementation science). We hope that this statement is sufficiently for you. Additionally we have added the following sentence in the manuscript methods section:

“The updated CFIR based on used feedback, as published in 2022, was used.”

**Reviewer #2: Comments to the Author**
Thank you for the opportunity to review this manuscript. The manuscript reports findings of the Implementation of Pelvic Floor Rehabilitation after rectal cancer surgery: A qualitative study guided by the Consolidated Framework for Implementation Research (CFIR). This is a well conducted qualitative study in an important clinical area. The authors describe a robust, well-designed study and have reported comprehensive results. That said, the manuscript in its present form is accepted without any general comments for consideration. Please could the authors include:

**Question/request:** - In the introduction, please add the definition of rectal cancer, prevalence, sex, and age that are most involved. Additionally, explain the low anterior resection syndrome and the sequences.

**Answer:** We have adjusted the introduction section and added the requested items.

Please see the revised manuscript. For the selection that is revised, see text below:

*“Rectal cancer is a disease in which malignant cells form in the tissues of the rectum. It has a lifetime probability of around 5% and the majority of disease occurs in people older than 50 with a slight predominance in males. Low anterior resection (LAR) is frequently used form of sphincter-preserving surgery and has good oncological outcomes. However, up to 90% of patients after LAR suffer from anorectal dysfunction, which is collectively called the low anterior resection syndrome (LARS). The symptoms associated with this syndrome, which include fecal incontinence, fragmentation, and clustering, have a significant impact on patient’s physical, mental and social functioning.”*

**Question/request: - Arrange for a review of the written English as there are some instances where the quality of the writing could be improved.**

**Answer: We performed an additional written English check and improved the text.**

**Question/request:** Please add the name of the ethics committee that provided approval for the study and the date and year approval was obtained in the method section.

**Answer:** We have adjusted the method section and added the requested items.

Please see the revised manuscript. For the selection that is revised, see text below:

*“The FORCE trial was registered in the Netherlands Trial Registration (NTR5469) in 09-2015, and was approved by the Medical Ethics Committee Arnhem/Nijmegen in 05-2027 (NL59799.091.16).”*

**Reviewer #3:** 

**Abstract:
Question/request:** 1. In line 38, Conclusion should be written based on the obtained results. Your results have not been written with certainty.

**Answer:** Dear reviewer 3, it is not clear to me/us in what way we should revise our manuscript based on your statement. Can you perhaps rephrase the question? We are more than happy to address the raised questions.

**Question/request:** 2. In keywords quality of life should be deleted.

**Answer:** We have deleted the keyword Quality of life.

**Question/request:** 3. In line 30 delete mixed from qualitative research.

**Answer:** We have deleted the requested word.

**Methods**

**Question/request:** 4. Explain how do deepen responses post interview guide questions?

**Answer:** Dear reviewer 3, again it is not completely clear to us what you mean by the abovementioned question. After carefully studying the methods section, is think that you are aiming at the section cited below:

*The focus groups aimed to identify implementation barriers and facilitate consensus among participants regarding the findings of the individual interviews by encouraging the participants to discuss the topics amongst themselves*

The interviews (one-on-one) were held before the focus groups. If many questions were to arise from the individual interviews, than the focus groups were used to facilitate consensus (based on the questions that were derived from the interviews).

We hope that this answers your question sufficiently.

**Question/request:** 5. In method part, how do you ensure maximum variation, confirmability, trustworthiness of your study?
**Answer:** Thank you for this question. The maximum variation was ensured by exploring all the possible perspectives (physiotherapists, referring medical experts, and patients who participated in the intervention themselves). To go a step further and ensure maximum variation within the patients that participated (to prevent selection bias; so that only the patients with success from the intervention would present their opinions in this follow-up study); we randomly selected patients from all ranges of outcome effects (those with a good response to therapy, those with no change, those with deterioration of function over time). Therefore, we are convinced that we did everything in our power to ensure maximum variation.

Regarding confirmability; data were checked and rechecked throughout data collection and analysis. Not two, but three reviewers were used to ensure that results would likely be repeatable by others.

Regarding trustworthiness; we can only report an the degree of confidence in data, interpretation and methods as described in our manuscript to ensure the quality and therefore trustworthiness of this study. In the most ideal situation, there would be multiple trials that could evaluate their intervention using CFIR and than compare the results in order to enhance trustworthiness. However, since this is the first RCT within this field of research that performed an qualitative analysis with CFIR; this is not possible.

We hope that this answers your questions sufficiently.

**Data analysis**
**Question/request:** How do you analyze the data and extract codes?

**Answer:**

After transcribing the audio recordings, major themes (codes) were identified. These analyses was done by multiple members of the author group. Afterwards, we organized the data by question and theme by highlighting questions, making notes and adding comments throughout the transcript. These quotes are also used in the tables included in the manuscript, to enhance certain statements. At last, disagreements were solved by means of an extra researcher who evaluated the transcripts.

Since we believe that this is all summarized in our data analysis section, we have not made any changes. We hope that this answers your question sufficiently.
